# Advancing Control of Cholera in the Interest of the Most Vulnerable in our Global Society

**DOI:** 10.1093/infdis/jiy458

**Published:** 2018-09-01

**Authors:** Louise C Ivers

**Affiliations:** Center for Global Health, Massachusetts General Hospital, and Department of Global Health and Social Medicine, Harvard Medical School, Boston

New approaches are needed to address the suffering and death caused by cholera. Having shaped populations for centuries, *Vibrio cholerae* is still responsible for completely preventable morbidity and mortality, the exact extent of which is not completely known. Estimated reports of cases are 2.9 million and 95000 deaths per year, but these are considered to be underestimates [1]. A number of countries do not report cholera cases at all, even when the disease is confirmed to occur. The stigma of the disease for individuals, and of epidemics of the disease for countries, remains high, with fear of impacts on trade and tourism, and perhaps also political implications for those that have failed to prevent and adequately protect their constituents. Biologically, the risk of infection and disease with cholera varies based on an individual’s prior exposure and existing level of immunity, but social determinants are the primary influencers of illness and of death from cholera. Those without clean water, with lack of access to soap and sanitation, the displaced, the food insecure and the impoverished are most at risk of being infected, and when they become ill, are most likely to die.

Cholera occurs when human feces have contaminated food or drinking water, and a lack of safe water is the biggest risk factor for outbreaks—a risk that is experienced by at least 2 billion people on the planet [2]. As a global community, we seem far too tolerant of the violation of the human right to clean water, essential as it is not just to preventing cholera but also to the control of other diarrheal diseases, essential for good nutrition and healthy growth, and critical for human dignity. Water insecurity places families in precarious situations, negatively influencing their choices and contributing substantially to ill health. In discussing Sustainable Development Goal 6—“Ensure access to water and sanitation for all”—United Nations literature states that “Clean water is a basic human need, and one that should be easily accessible to all” [3], yet despite the fact that it should be “easy,” we have not yet delivered. This is not really the result of technical challenges—though there are likely some. Rather, it is predominantly due to a lack of will to prioritize the issue and to invest the resources to get the job done. This is inexcusable.

Meanwhile, as cholera cases continue to occur, we know what to do to save lives now, and there are opportunities to improve our surveillance, to reduce case-fatality rates, and to prevent transmission of cholera through individual-level and household-level interventions. In severe cases, as vomiting and profuse watery diarrhea ensue, the majority of deaths are related to hypovolemic shock. Prompt treatment with oral rehydration therapy (ORT) is lifesaving. ORT is simple, safe and inexpensive, and estimated to have saved millions of lives since its creation in the 1960s. Providing ORT, however, requires that a population at risk interacts with a health system in some way—even a system of modest quality can provide the necessities—through educated family members, equipped community health workers, or facility-based care.

Some patients need other interventions as well, such as intravenous fluids, zinc, and antibiotics, though case-fatality rates from cholera are still far too high [4], a signal of the failure of health systems and a marker of the precarious situation in which many patients with cholera are living. A case-fatality rate <1% is often cited as a benchmark goal for outbreak response, but although that represents a substantial improvement from the pre-ORT era, it shows to some degree how our expectations have stalled on what it is possible for us to achieve. Some institutions, for example, in Bangladesh [5] have shown far lower case-fatality rates, even in limited-resource situations. Finding and treating patients earlier and a better understanding of the care of special populations, including pregnant women, people living with human immunodeficiency virus, prison populations, and children with malnutrition, are key to reducing mortality rates further. So is investing in research on patient care; there has been some research, but not enough, on the care of particularly vulnerable groups, and even for some fundamental questions, such as the most appropriate use of antibiotics in treating cholera, we lack sufficient evidence to make definitive recommendations [6].

An ambitious multisectoral approach to cholera control and prevention was launched by the Global Task Force for Cholera Control and Prevention in 2018, with the goal of reducing cholera deaths by 90% by 2030 [7]. In a resolution proposed by Haiti and Zambia at the 71st World Health Assembly, health leaders of the world’s nations agreed on making cholera a priority and endorsed this reinvigorated multisectoral approach to addressing it. Many authors of articles in the current supplement (along with other academics, public health practitioners, nongovernmental organization staff, public servants, and frontline health workers) are part of a revitalized effort to make that goal a reality, and they bring their experience to this collection.

This supplement collates articles on the epidemiology of cholera globally, with a discussion of the “Roadmap to 2030” [8], as well as articles on the state of science in various aspects that are key to prevention and control efforts, including reviews on the immunology of cholera and prospects for future vaccines [9], what we know and still need to learn about individual and household risk factors for cholera [10], and the effectiveness of household water treatment in cholera control [11]. These articles are followed by a series of country-specific perspectives from health workers, program implementers, and physician scientists working at the front lines of epidemic and endemic cholera in Zambia, Bangladesh, Zanzibar, and Haiti, as well as a perspective by Médecins sans Frontières on the challenges and use of oral cholera vaccines in their work responding to persons afflicted by crisis around the world [12].

To move forward on cholera control globally, we should accept that lifesaving—albeit sometimes short-term—interventions must occur contemporaneously with the longer-term work of realizing the right to safe water and sanitation. “Long-term” can mean as short as a decade—cholera transmission was largely eliminated from Latin America in the 1990s [13]—and it should not be used as a euphemism for “indefinite.” Constrained by meager resources and socialized for scarcity, public health experts often spend too much time debating either/or approaches when more than one may be needed for the biggest impact. We might also use our platforms as physicians and public health providers to more wholly interrogate constructs of scarcity, arguing in favor of expanding available resources for public health interventions across the board [14].

Of course, one way to eliminate cholera as a global concern is to completely transform global development. Large-scale investment in universal health coverage, public water and sanitation, and universal access to education would pay social returns far above and beyond cholera and diarrheal disease, and would transform societies. Cholera is in many ways a bellwether for these failing systems, and its occurrence should be a call to action. At times, however, our biggest limitation is our collective failure of imagination as to what we can do for the poor and vulnerable in society. In this sense, the ambitious “Roadmap to 2030” is a welcome reprieve. Cholera has been a forcing function in science and public health for 2 centuries—prompting scientific discoveries, fostering ingenuity and creativity in immunology and vaccinology, and inspiring the foundation of public health principles. Let us not stop now. We can and must work collectively to deliver on the promise to end cholera.

## Note

***Potential conflicts of interest.*** Author certifies no potential conflicts of interest. The author has submitted the ICMJE Form for Disclosure of Potential Conflicts of Interest. Conflicts that the editors consider relevant to the content of the manuscript have been disclosed.
